# TSC1 Affects the Process of Renal Ischemia-Reperfusion Injury by Controlling Macrophage Polarization

**DOI:** 10.3389/fimmu.2021.637335

**Published:** 2021-03-09

**Authors:** Xiao Hu, Yanan Xu, Zhaoqi Zhang, Zuofu Tang, Jinhua Zhang, You Luo, Weiming Deng, Zhanwen Dong, Yong Zhao, Ning Na

**Affiliations:** ^1^Department of Kidney Transplantation, The Third Affiliated Hospital of Sun Yat-sen University, Guangzhou, China; ^2^State Key Laboratory of Membrane Biology, Institute of Zoology, Chinese Academy of Sciences, Beijing, China; ^3^Savaid Medical School, University of Chinese Academy of Sciences, Beijing, China; ^4^Institute for Stem Cell and Regeneration, Chinese Academy of Sciences, Beijing, China

**Keywords:** kidney, ischemia-reperfusion (IR), macrophage polarization, fibrosis, tuberous sclerosis complex 1 (TSC1)

## Abstract

Renal ischemia-reperfusion injury (IRI) contributes to acute kidney injury (AKI), increases morbidity and mortality, and is a significant risk factor for chronic kidney disease (CKD). Macrophage infiltration is a common feature after renal IRI, and infiltrating macrophages can be polarized into the following two distinct types: M1 macrophages, i.e., classically activated macrophages, which can not only inhibit infection but also accelerate renal injury, and M2 macrophages, i.e., alternatively activated macrophages, which have a repair phenotype that can promote wound healing and subsequent fibrosis. The role of TSC1, which is a negative regulator of mTOR signaling that regulates macrophage polarization in inflammation-linked diseases, has been well documented, but whether TSC1 contributes to macrophage polarization in the process of IRI is still unknown. Here, by using a mouse model of renal ischemia-reperfusion, we found that myeloid cell-specific TSC1 knockout mice (termed Lyz-TSC1 cKO mice) had higher serum creatinine levels, more severe histological damage, and greater proinflammatory cytokine production than wild-type (WT) mice during the early phase after renal ischemia-reperfusion. Furthermore, the Lyz-TSC1 cKO mice showed attenuated renal fibrosis during the repair phase of IRI with decreased levels of M2 markers on macrophages in the operated kidneys, which was further confirmed in a cell model of hypoxia-reoxygenation (H/R) *in vitro*. Mechanistically, by using RNA sequencing of sorted renal macrophages, we found that the expression of most M1-related genes was upregulated in the Lyz-TSC1 cKO group (Supplemental Table 1) during the early phase. However, C/EBPβ and CD206 expression was decreased during the repair phase compared to in the WT group. Overall, our findings demonstrate that the expression of TSC1 in macrophages contributes to the whole process of IRI but serves as an inflammation suppressor during the early phase and a fibrosis promoter during the repair phase.

## Introduction

Renal ischemia-reperfusion injury is a major risk factor affecting the prognosis of AKI and the functional recovery and long-term survival of grafts after renal transplantation (1). Innate and adaptive immune cells, such as macrophages, dendritic cells, neutrophils, and lymphocytes, are involved in the pathogenesis of renal injury after ischemia-reperfusion injury (IRI) (2). Specifically, macrophages, which are primary components of the phagocytic system, aggravate damage during the initial stage of reperfusion but promote tubular repair and long-term renal fibrosis during the late stage after IRI (3). During the early phase, monocytes adhere to the vasa recta 2 h after IRI, and most macrophage recruitment occurs surrounding postcapillary venules (4). Then, proinflammatory monocytes/macrophages are thought to mediate tubular injury by releasing several important cytokines (such as IL-6, TNF-α, and IL-12) and nitric oxide (NO) by iNOS (3). Among all cell types associated with renal fibrogenesis, macrophages can promote the progression of renal fibrosis as a driving force; CD206^+^ M2 macrophages, especially those derived from bone marrow cells that can directly contribute to renal fibrosis, are strongly involved in renal fibrosis (5). In the injured kidney, macrophages from bone marrow cells can differentiate into α-SMA^+^ myofibroblasts termed macrophage-to-myofibroblast transition (MMT), which contribute to pathogenic collagen production during tissue fibrosis (6–8). The CD206+ α-SMA+ cells co-expressing suggests that macrophages that undergo MMT are predominantly M2 (5, 9). Moreover, some cytokines and growth factors secreted by macrophages, such as TGF-β1, IL-10, and matrix metalloproteinase (MMP), can contribute to fibrosis (10). In addition, it has been confirmed in various disease models that transforming growth factor-β (TGF-β) is a main cause of fibrosis in most forms of chronic kidney disease (CKD) (11). IL-10 is considered an anti-inflammatory and antifibrotic cytokine, but paradoxically, IL-10 appears to affect profibrotic signaling pathways that are unique to the kidney (12). Barbarin et al. reported that IL-10 inhibits the infiltration of inflammatory cells during the early stage of fibrosis but promoted fibrosis during the later stage (13).

Macrophages can differentiate into distinct phenotypes when stimulated by certain cytokines or exposed to different microenvironments (14). M1 macrophages are induced by exposure to IFNγ, LPS, TNFα, or GM-CSF and express proinflammatory cytokines, such as IL-1β, TNFα, and IL-6, while M2 macrophages resulting from IL-4/IL-13 stimulation produce anti-inflammatory factors, including IL-10 and TGFβ (15, 16). Several studies have shown that renal tubular cells participate in the activation and polarization of macrophages by producing macrophage growth factors, such as macrophage colony-stimulating factor (M-CSF), granulocyte macrophage colony-stimulating factor (GM-CSF), and IL-34 (17–20). In coculture with tubular epithelial cells, macrophages activate and upregulate the expression of the alternative activation markers CD206 and Arginase-1 (Arg-1) (21). These conclusions imply that macrophages contribute to renal ischemia-reperfusion injury and subsequent recovery *via* different phenotypic polarizations.

The tuberous sclerosis complex (TSC) comprises TSC1, TSC2, and TBC1D7 (22). The mechanistic target of rapamycin (mTOR) is a serine/threonine protein kinase belonging to the PI3K-related kinase (PIKK) family that forms the catalytic subunit of two protein complexes, i.e., mTOR complex 1 (mTORC1) and mTOR complex 1 (mTORC2); the TSC1 and TSC2 proteins form a heterodimeric complex that acts as a functional unit that inhibits mTOR as the key negative regulator of mTORC1 signaling (23). Using an acute endotoxin shock model, our previous study revealed that TSC1 inhibits M1 polarization by suppressing the Ras GTPase–Raf1–MEK–ERK pathway in an mTOR-independent manner while promoting M2 properties by mTOR-dependent CCAAT/enhancer-binding protein-b (C/EBP-β) pathways (24).

This study demonstrates that *TSC1* deficiency in macrophages promotes M1 polarization to aggravate kidney dysfunction during the early phase of renal IRI and attenuates renal fibrosis during the repair stage resulting from reduced M2 polarization. Therefore, these findings demonstrate that TSC1 controls macrophage polarization to affect the whole process of renal ischemia-reperfusion injury.

## Methods

### Mice

Myeloid cell-specific TSC1 conditional knockout mice (C57BL/6 background) were obtained by crossing TSC1^loxp/loxp^ mice with mice expressing Cre recombinase under the control of the lysozyme promoter (LysMCre), and WT littermates were used as controls. The TSC1^loxp/loxp^ mice were kindly provided by Dr. Hongbin Zhang (Institute of Basic Medical Sciences and School of Basic Medicine, Peking Union Medical College, and Chinese Academy of Medical Sciences, Beijing, China) (25, 26). The LysMCre mice were generous gifts from Dr. Lianfeng Zhang, Key Laboratory of Human Diseases Comparative Medicine, Ministry of Health, Institute of Laboratory Animal Science, CAMS & PUMC (27). All mice were maintained in the specific pathogen-free Laboratory Animal Centre of the Institute of Zoology, Beijing, China. All studies were performed according to the institutional guidelines and approved by the research ethics committee (IRB: [2020]02-166) of the Third Affiliated Hospital of Sun Yat-sen University.

### IRI Model

Male C57BL/6 mice (aged 6–8 weeks; weight 18–22 g) were anesthetized using isoflurane (2% for induction and 1.5% for maintenance) on a 36°C warming pad using a rectal probe. Non-damaged microvascular clamps (AESCULAP YASARGIL, Cat: FT222T) were used to block the bilateral renal pedicles for 30 min. The peritoneum and skin were closed in turn with 5-0 silk threads. One milliliter of saline was injected *via* the intraperitoneal route to prevent dehydration. All mice were kept under 12-h light/dark cycles, and normal food was provided *ad libitum*. The mice were euthanized, and the kidneys were collected on days 1, 4, 7, 14, and 21 after IRI. The kidney tissue was harvested for the histology, immunohistochemistry, and immunofluorescence analyses, RNA seq and RNA extraction after fluorescence-activated cell sorting (FACS).

### BMDMs and Adoptive Transfer

After separating the tibia, femur, and ilium of the mice, the cells in the bone marrow were collected with a syringe filled with Dulbecco’s modified Eagle medium (DMEM). The bone marrow cells were cultured in DMEM supplemented with 10% fetal bovine serum (FBS) and 10 ng/ml mouse macrophage colony-stimulating factor (M-CSF) for 7 days to acquire bone marrow-derived macrophages (BMDMs). Then, the harvested BMDMs were labeled by CFSE (MedChemExpress, Cat No.: HY-D0938) as previously reported (16). CFSE-labeled WT/Lyz-TSC1 cKO BMDMs (1 × 107) were intravenously injected into WT mice 2 h before kidney IRI.

### Hypoxia-Reoxygenation (H/R) Injury in RTECs *In Vitro*

Renal tubular epithelial cells (RTECs, BeNa Culture, Cat: BNCC340198) were cultured in DMEM supplemented with 5% FBS, penicillin (100 U/ml), and streptomycin (100 μg/ml) until they reached 90% confluence. Then, the cells were exposed to serum-free medium and cultured in a hypoxic incubator (1% O2, 94% N2, and 5% CO2) for 4 h at 37°C to induce hypoxic injury. Then, the cells were returned to 5% CO2 and 95% air for reoxygenation. The supernatants were harvested at 24 h after centrifugation to remove cell impurities. The BMDMs from the WT and Lyz-TSC1 cKO mice were treated with CM supplemented with 5% FBS, penicillin (100 U/ml), and streptomycin (100 μg/ml) for 24–48 h and were collected for RNA extraction.

### Kidney Histology and Immunohistochemical and Immunofluorescence Staining

The kidney samples were embedded in paraffin. Sections at a thickness of 4 μm were used for the hematoxylin and eosin (H&E), Masson staining, and Sirius Red staining following standard protocols. Renal tubular damage was graded on a five-level scale according to the extent of necrosis in the proximal tubules as described by Jablonski et al. (28). All assessments were performed by two investigators blinded to the experimental conditions. The positively stained tissue relative to the selected field was quantified by ImageJ (National Institutes of Health, Bethesda, MD, USA). For IHC experiments, α-SMA, Collagen III, fibronectin, and KIM-1 were detected by immunohistochemistry using primary monoclonal antibodies against α-SMA (Servicebio, Cat: GB13044), Collagen III (Servicebio, Cat: GB13023-2), fibronectin (Servicebio, Cat: GB13091), and primary polyclonal antibodies against KIM-1(Sigma, Cat: SAB3500252-100ug). F4/80 and CD206 were detected by immunofluorescence using primary monoclonal antibodies against F4/80 (Abcam, Cat: ab6640) and primary polyclonal antibodies against CD206 (Abcam, Cat: Ab64693). CD206 and α-SMA were also detected by immunofluorescence using amCD206-FITC (Biolengend, Cat: 141703) and primary monoclonal antibodies against α-SMA (Abclonal, Cat: A17910). Fluorescent images were acquired using a Ti-E A1R+STORM confocal microscope.

### Assessment of Renal Function

Serum creatinine was measured to assess renal function and analyzed by an AU5811 analyzer (Beckman Coulter) using the enzymatic assay method (DONGOU, Cat: 59400377071).

### Flow Cytometry

The recipient mice were sacrificed and perfused with 20 ml 0.9% NaCl solution containing 125 U/ml heparin sodium *in situ*. The kidneys were acquired and grinded. A single-cell suspension was digested at 37°C for 30 min in 1640 medium containing 1 mg/ml type IV collagenase (Sigma, Cat: C5138-5 g) and 20 U/ml DNase I (Sigma, Cat: D5025-150 KU). Then, the cells were stained with antibodies (including CD45 [30-F11], F4/80 [BM8], CD11b [M1/70] [from eBioscience {San Diego, CA, USA}], and CD206 [C068C2] [from BD Biosciences {San Jose, CA, USA}]) against surface antigens for 30 min at 4℃. For FACS, the cells were stained with the following antibodies: anti-F4/80 (BM8) (eBioscience), anti-CD45.2 (clone 104), and anti-CD11b (M1/70) (from eBioscience). Then, the labeled cells were sorted by a MoFlo XDP sorter (Beckman). All flow cytometry data were obtained with an LSRFortessa™ X-20 instrument (BD Biosciences, CA, USA) and analyzed with FlowJo (Treestar, OR, USA).

### RNA-Seq Analysis

The macrophages were sorted by CD45^+^F4/80^+^CD11b^+^ in the kidneys from the WT and Lyz-TSC1 cKO mice at 24 h and 2 weeks after IRI using a MoFlo XDP cell sorter (BeckMan Coulter, Brea, CA, USA). All samples were collected in tubes containing lysis components and ribonuclease inhibitors. All RNA extraction, library preparation, and sequencing were performed by Annoroad Gene Tech. (Beijing) Co., Ltd. Then, amplification was carried out by the Smart-Seq2 method. An oligo-dT primer was introduced to the reverse transcription reaction for the first-strand cDNA synthesis, followed by PCR amplification to enrich the cDNA and magbead purification step to clean the production. Then, the cDNA production was checked by Qubit^®^ 3.0 Fluorometer and Agilent 2100 Bioanalyzer to ensure the expected production with a length of approximately 1~2 kbp. Then, the cDNA was randomly sheared by ultrasonication for the Illumina library preparation protocol, including DNA fragmentation, end repair, 3’ end A-tailing, adapter ligation, PCR amplification, and library validation. After the library preparation, a PerkinElmer LabChip^®^ GX Touch and Step OnePlus™ Real-Time PCR System was used for the library quality inspection. Then, the qualified libraries were loaded onto the Illumina HiSeq platform for the PE150 sequencing. We downloaded macrophage polarization data from the NCBI GEO database (GSE107776).

For both the downloaded data and our data, we used the mapping software HISAT2 to map the reads to the mouse mm10 reference genome and StringTie to construct transcripts independently for each cell (29). DEseq2 was used to identify the differentially expressed genes between the WT and TSC1-deficient macrophages (30). We considered adjp < 0.05 and |log2(fold change)| > 1 indicative of a significant difference in the two cell thresholds. Gene Ontology (GO) functional annotation and KEGG pathway analysis were performed to analyze all differential genes using the DAVID Bioinformatics Resources 6.8 online search tool (https://david.ncifcrf.gov/) and the Kobas online search tool (http://kobas.cbi.pku.edu.cn/). The functional protein network analysis was performed by String (https://string-db.org/) and shown by Cytoscape software (31).

### Quantitative PCR Analysis

Kidney RNA, FACS-sorted cell RNA, or cultured cell RNA was extracted with TRIzol Reagent (Invitrogen, Carlsbad, CA, USA) or E.Z.N.A. Total RNA kit I (#R6834) and reverse transcribed (Bio-Rad). The RNA was quantified using a Take3 Gen5 spectrophotometer (BioTek, Winooski, VT). The gene expression analysis was determined by quantitative real-time PCR (iTaq Universal Sybr Green Supermix; Bio-Rad) using CFX96 (Bio-Rad) and normalized to the expression level of the housekeeping gene hypoxanthine phosphoribosyltransferase.

### Statistical Analysis

All results are expressed as the mean ± SEM. GraphPad Prism software (GraphPad Inc., San Diego, CA, USA) was used to analyze the data. Unpaired t-tests and analysis of variance were performed, and P < 0.05 was considered indicative of statistical significance.

## Results

### Elevated Injury Was Caused by Increased Proinflammatory Polarization of *TSC1* Knockout Macrophages in the Kidney During the Early Phase

**Figure 1:**
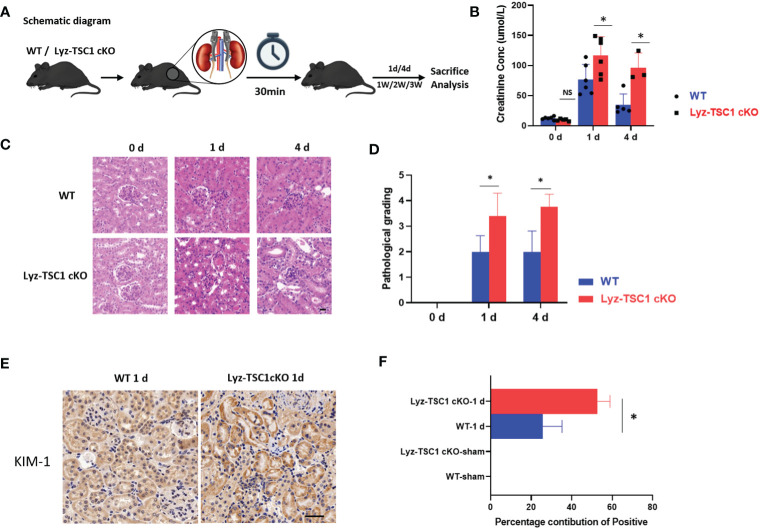
*TSC1* depletion in macrophage aggravated mice IR-induced tubular injury. **(A)** Schematic diagram. **(B)** Serum creatinine levels in wild-type (WT) and Lyz-TSC1 cKO mice at baseline (0) and 1 and 4 days post-IRI. **(C)** Representative H&E-stained renal sections of WT and Lyz-TSC1 cKO mice. Scale bar: 20 µm. **(D)** Quantification of H&E staining corresponding to the WT and Lyz-TSC1 cKO mice shown in **(C)**. **(E)** Representative Kidney Injury Molecule 1 (KIM-1) immunohistochemistry in renal sections from WT and KO mice on day 1 post-IRI (×200). **(F)** Quantification of KIM-1 immunohistochemistry-stained murine renal cortical sections on day 0 and day 1 after IRI. In **(B**, **D**, **F)**, the data are shown as the mean ± SD and were analyzed by an unpaired two-tailed Student’s t-test (n ≥ 3). *P < 0.05; **P < 0.01; NS, not significant.

To explore the role of TSC1 expression in macrophages during the process of renal IRI, a standardized model of renal IRI was established in this study by blocking the bilateral renal pedicles for 30 min. The kidneys were obtained on days 1, 4, 7, 14, and 21 after IRI (**Figure 1A**). During the early phase after reperfusion, the Lyz-TSC1 cKO mice showed aggravated kidney dysfunction, and significantly higher serum creatinine levels were observed in the Lyz-TSC1 cKO mice 1 and 3 days after injury (**Figure 1B**). In addition, the renal tubular injury scores were assessed 1 and 3 days after reperfusion by H&E staining (**Figure 1C**). The renal tubular injury scores of the Lyz-TSC1 cKO mice were significantly higher than those of the wild-type mice, suggesting accentuation of tubular injury in the Lyz-TSC1 cKO mice (**Figure 1D**). Kidney injury molecule-1 (Kim-1) is an acute kidney injury (AKI) biomarker that is always upregulated during the early phase of IRI. In the sham-operated mice, the immunostaining of Kim-1 could not be detected in the kidney. However, the positively stained kidney area was significantly increased in the Lyz-TSC1 cKO mice compared to that in the WT kidneys (**Figures 1E, F**). These results indicate that the AKI induced by ischemia-reperfusion in the Lyz-TSC1 cKO mice is more severe than that in the control mice.

**Figure 2:**
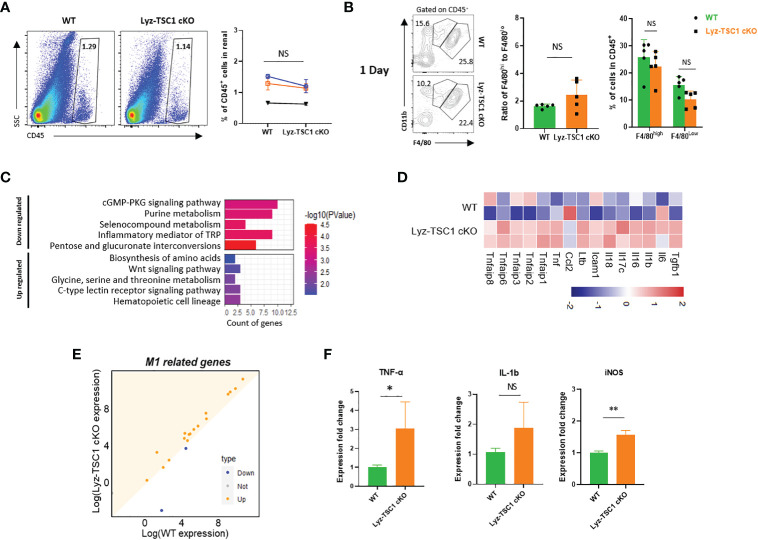
Increased M1 polarization of *TSC1* knockout macrophages during the early phase. **(A)** Inflammatory cell infiltration and percentage of CD45^+^ cells in WT and Lyz-TSC1 cKO kidneys on day 1 after reperfusion. **(B)** F4/80^high^ macrophages and F4/80^low^ macrophages from WT and Lyz-TSC1 cKO mice gated on CD45^+^ cells. **(C)** Histogram of the top 10 most up- and downregulated KEGG pathways. **(D)** Dot plot of M1-related genes. The orange color represents the upregulated genes in the Lyz-TSC1 cKO macrophages. **(E)** Heatmap of up- and downregulated M1-related genes in the WT and Lyz-TSC1 cKO groups. **(F)** Relative mRNA expression of interleukin -1β (IL-1β), tumor necrosis factor-α (TNF-α), and iNOS in kidney macrophages sorted from WT and Lyz-TSC1 cKO mice 24 h after IRI. In **(A**, **B**, **F)**, the data are shown as the mean ± SD and were analyzed by an unpaired two-tailed Student’s t-test (n ≥ 3). *P < 0.05; **P < 0.01; NS, not significant.

Renal interstitial cell infiltration after I/R mainly involves monocytes/macrophages and is a vital indicator of inflammation after IR injury (32). However, by conducting a flow cytometry analysis, we found that the levels of renal total leukocytes (CD45^+^) in the WT and Lyz-TSC1 cKO mice were comparable before and 1 day after the ischemia-reperfusion operation (**Supplementary Figure 1A**, **Figure 2A**). Previous studies have shown that F4/80^low^CD11b^+^ cells are M1-like macrophages, while F4/80^high^CD11b^+^ cells are M2-like macrophages (33, 34). In our study, the ratios of F4/80^high^ macrophages and F4/80^low^ cells in the WT and Lyz-TSC1 cKO mice gated on CD45^+^ were also comparable before and 1 day after reperfusion (**Supplementary Figure 1B**, **Figure 2B**). To evaluate the infiltration ability of TSC1 cKO macrophages, we labeled the WT and Lyz-TSC1 cKO mouse-derived BMDMs with CFSE and adoptively transferred them to WT recipients 2 h before kidney IRI (**Supplementary Figure 2A**). After 24 h of reperfusion, we obtained the kidneys to trail the transferred cells by flow cytometry. The data show that there was no statistically significant difference in the number of CFSE-labeled WT BMDMs and TSC1 cKO BMDMs that infiltrated the kidneys (**Supplementary Figures 2B, C**). These data indicate that the elevated renal IRI in the Lyz-TSC1 cKO mice was not caused by increased infiltration of inflammatory macrophages during the initial stage.

To ensure functional shifts of macrophages during the early stage of reperfusion, we sorted the kidney macrophages (CD45^+^F4/80^+^CD11b^+^) in the WT and Lyz-TSC1 cKO mice 24 h after reperfusion and performed RNA sequencing. Although the pathways related to macrophage polarization were not obvious among the most enriched pathways of DEGs, most M1-related genes (*Tgfb1*, *Il6*, *Il1b*, *Il16*, *Il17c*, *Il18*, *Icam1*, *Ltb*, *Ccl2*, *Tnf*, *Tnfaip1*, *Tnfaip2*, *Tnfaip3*, *Tnfaip6*, and *Tnfaip8*) in the Lyz-TSC1 cKO group were upregulated compared to those in the WT group (**Figures 2C–E**) (35). To further verify these results, we performed a quantitative real-time PCR analysis of FACS-sorted macrophages from kidneys 24 h post-IRI. The results show that the macrophages sorted from the kidneys of the Lyz-TSC1 cKO mice had increased expression of interleukin-1β (IL-1β), tumor necrosis factor-a (TNF-a), and iNOS, which are hallmarks of M1 polarization (**Figure 2F**) (34). These data demonstrate that *TSC1* deficiency promoted renal macrophage inflammatory M1 polarization to enhance inflammation and aggravate early tubular epithelial injury after ischemia-reperfusion during the early phase.

### Conditional Depletion of *TSC1* in Macrophages Reduced Renal Fibrosis After IRI

**Figure 3:**
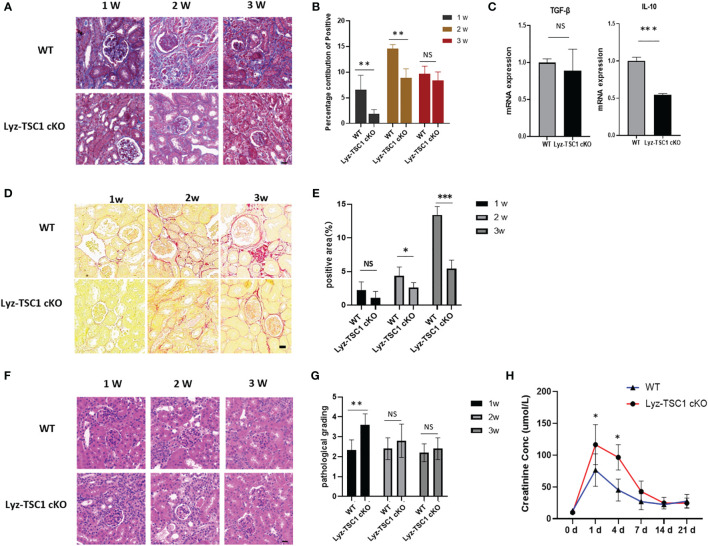
Decreased renal fibrosis after IRI in Lyz-TSC1 cKO mice. **(A)** Representative images of Masson’s trichrome staining of WT and Lyz-TSC1 cKO kidneys on days 7, 14, and 21 after reperfusion. Scale bar: 20 µm. **(B)** Quantification of Masson’s trichrome staining corresponding to the WT and Lyz-TSC1 cKO mice shown in **(A)**. **(C)** Fibrosis-related cytokines (transforming growth factor-β [TGF-β] and IL-10) mRNA relative expression in kidneys from WT or Lyz-TSC1 cKO mice on day 14 after reperfusion. **(D)** Representative Sirius Red-stained renal sections of WT and Lyz-TSC1 cKO mice. Scale bar: 20 µm. **(E)** Quantification of Sirius Red staining corresponding to the WT and Lyz-TSC1 cKO mice shown in **(D)**. **(F)** Representative images of HE staining of WT and Lyz-TSC1 cKO kidneys on days 7, 14, and 21 after reperfusion. Scale bar: 20 µm. **(G)** Renal tubular injury scores of H&E staining corresponding to the WT and Lyz-TSC1 cKO mice shown in **(F)**. **(H)** Serum creatinine levels in WT and Lyz-TSC1 cKO mice on days 7, 14, and 21 after ischemia-reperfusion. In **(B**, **C**, **E**, **G**, **H)**, the data are shown as the mean ± SD and were analyzed by an unpaired two-tailed Student’s t-test (n ≥ 3). *P < 0.05; **P < 0.01; NS, not significant.

We also explored the performance in the two groups during the late stage of renal ischemia-reperfusion. We performed Masson’s trichrome staining to assess renal fibrosis 1, 2, and 3 weeks after reperfusion (**Figure 3A**). Interestingly, the Masson staining of the Lyz-TSC1 cKO group showed decreased collagen deposition compared to that in the WT group, especially at 2 weeks (**Figure 3B**). As fibrosis-related cytokines, we examined the transcription of transforming growth factor-β (TGF-β) and IL-10 in the kidneys 14 days after ischemia-reperfusion. Compared to the control littermates, much lower IL-10 levels were detected in the knockouts, while the level of TGF-β did not significantly differ between the two groups (**Figure 3C**). The positive area of Sirius Red was increased during this process, with a sharp increase at day 21 (**Figures 3D, E**). Collagens especially collagen III (COL 3), fibronectin (FN), alpha-SMA are metabolic balance-related genes and proteins of extracellular matrix (ECM) (36). Immunohistochemistry showed that Lyz-TSC1 CKO kidneys had a reduced deposition of collagen III, α-SMA, and fibronectin (FN), compared to WT kidneys (**Supplementary Figures 3A–F**). We also assessed the renal tubular injury scores of the two groups by H&E staining during the repair phase after IR (**Figure 3F**). The renal tubular damage score of the Lyz-TSC1 cKO mice tended to be slightly higher than that of the wild-type mice during the initial stage of restoration, but the degree of damage between the two groups did not continue to deteriorate during the late phase (**Figure 3G**), and serum creatinine showed a similar trend (**Figure 3H**). The serum creatinine levels in the Lyz-TSC1 cKO mice were significantly higher 7 days post-IRI and then rapidly returned to levels comparable to those in the WT mice. These results suggest that the deletion of *TSC1* in macrophages reduces kidney fibrosis after IRI in mice and prevents further deterioration of tubular injury to a certain extent.

### M2 Macrophages in the Kidneys of Lyz-TSC1 cKO Mice Are Significantly Reduced During the Ischemia-Reperfusion Repair Phase

**Figure 4:**
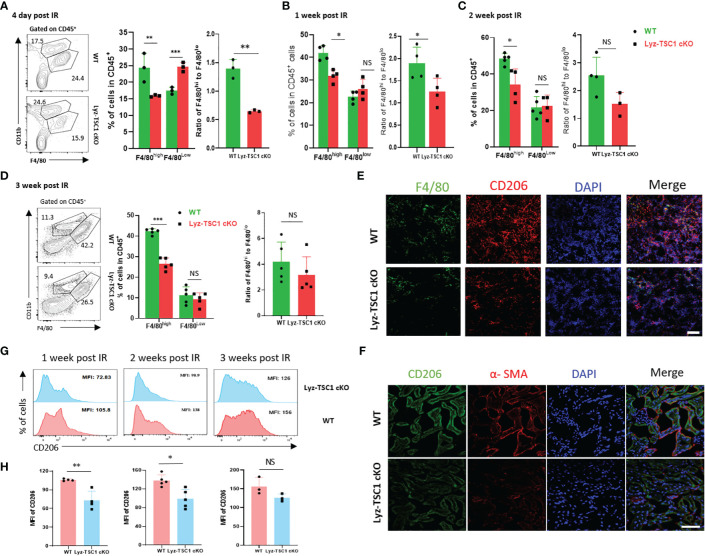
Decreased M2 polarization in Lyz-TSC1 cKO macrophages during the repair process of renal ischemia-reperfusion. **(A–D)** Left panel: representative dot plots of the proportion of F4/80^hi^ and F4/80^low^ macrophage subsets in CD45^+^ cells in the WT and Lyz-TSC1 cKO kidneys. Middle panel: quantification of the proportion of F4/80^hi^ and F4/80^low^ macrophage subsets in CD45^+^ cells. Right panel: ratio of F4/80^hi^ and F4/80^low^ macrophage subsets. **(A)** Day 4 post-IRI. **(B)** Day 7 post-IRI. **(C)** Day 14 post-IRI. **(D)** Day 21 post-IRI. **(E)** M2 macrophages were double-positive for F4/80 (green) and CD206 (red) by immunofluorescence in the WT and Lyz-TSC1 cKO kidneys on day 14. Scale bar: 50 µm. **(F)** Kidney sections were subjected to immunofluorescence staining for CD206 and a-SMA. Scale bar: 50 µm. **(G, H)** CD206 mean fluorescence intensity (MFI) in macrophages in WT and Lyz-TSC1 cKO mice kidney were analyzed after ischemia-reperfusion injury on days 7, 14, and 21. Each dot represents an individual mouse. In **(A–D, G, H)**, the data are shown as the mean ± SD and were analyzed by an unpaired two-tailed Student’s t-test (n ≥ 3). *P < 0.05; **P < 0.01; NS, not significant.

Macrophages (especially the CD206^+^ subset of M2 macrophages) are strongly associated with renal fibrosis and tubule repair in both human and experimental diseases (5, 21). Thus, we hypothesized that the depletion of *TSC1* in macrophages would reduce fibrosis because of the attenuated polarization to M2 after ischemia-reperfusion. To verify this hypothesis, we determined whether the F4/80^high^CD11b^+^ macrophages in the kidney from the Lyz-TSC1 cKO mice were decreased 7, 14, and 21 days after IRI. M2 macrophages (also termed alternatively activated) are anti-inflammatory and tend to promote injury repair and resolution of inflammation (3). The flow cytometry analysis showed that the Lyz-TSC1 cKO kidneys had significantly fewer F4/80^high^ (M2-like macrophages) macrophages and a lower ratio of F4/80^high^ to F4/80^low^ gating on CD45^+^ than the WT kidneys 7, 14, and 21 days after reperfusion (**Figures 4A–D**). Additionally, the levels of F4/80^high^ macrophages and the ratio of F4/80^high^ to F4/80^low^ in the WT and Lyz-TSC1 cKO mice were comparable before ischemia-reperfusion injury (**Supplementary Figure 1B**). CD206 (also known as mannose receptor) is a marker of M2 macrophages that is expressed in not only monocyte-derived macrophages (MDMs) but also tissue macrophages (37). To further assess M2 macrophage expression in the kidneys, we stained the kidney tissues and performed immunofluorescence. We costained the kidney tissues with antibodies against F4/80 and CD206 to identify M2 macrophages 14 days after IR. The number of stained M2 macrophages in the kidneys from the knockout mice was significantly lower than that in the control littermates (**Figure 4E**). Immunohistochemistry further showed that Lyz-TSC1 cKO group had a reduced co-expressing of CD206 and α-SMA, compared to WT group 14 days after IR (**Figure 4F**). Also, we determined the difference in CD206 expression between the WT group and the Lyz-TSC1 cKO group by a flow cytometry analysis. The mean fluorescence intensity (MFI) of CD206 in the WT and Lyz-TSC1 cKO mice was comparable before ischemia-reperfusion injury (**Supplementary Figure 1C**). Notably, CD206 expression in the renal macrophages in the Lyz-TSC1 cKO groups 7, 14, and 21 days after reperfusion was significantly lower than that in the WT group (**Figures 4G, H**). These results indicate that the decreased renal fibrosis after IRI in the Lyz-TSC1 cKO mice may result from the attenuated M2 polarization of *TSC1* cKO renal macrophages.

### The Defective Response of *TSC1* cKO Macrophages to CM Is Mediated by mTOR-C/EBPβ *In Vitro*

**Figure 5:**
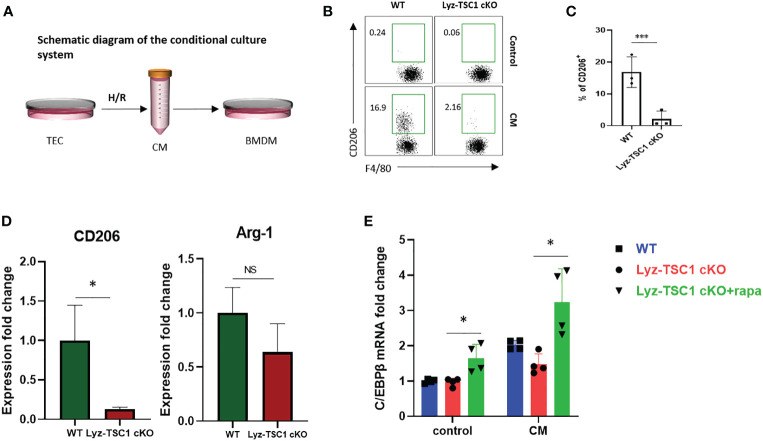
Defective polarization to conditional medium of *TSC1* cKO macrophages is mediated by mTOR-C/EBPβ *in vitro*. **(A)** Experimental design of the BMDMs from the WT and Lyz-TSC1 cKO mice, which were treated with CM from mouse renal TECs (mRTECs) cell line after hypoxia-reoxygenation (H/R) injury. **(B)** Representative dot plots of the CD206^+^ subset in the F4/80^+^ compartment in the WT or *TSC1* cKO BMDMs treated with conditional medium. **(C)** Quantification of the proportion of the CD206^+^ subset in F4/80^+^ macrophages. **(D)** Relative expression of CD206 and Arginase-1 (Arg-1) in WT or *TSC1* cKO BMDMs treated with CM. **(E)** Lyz-tsc1 cKO BMDMs were pretreated with Rapa (100 nM) for 1 h and then stimulated with CM for an additional 48 h. C/EBPβ expression in WT, Lyz-TSC1 cKO, and Lyz-TSC1 cKO treated with Rapa as determined by quantitative PCR after treatment with or without CM. In **(C**, **D)**, the data are shown as the mean ± SD and were analyzed by an unpaired two-tailed Student’s t-test (n ≥ 3). *P < 0.05; **P < 0.01; NS, not significant.

Tubular epithelial cells (TECs) play diverse roles in renal repair or progression to chronic kidney disease (CKD) by releasing bioactive mediators that drive fibrosis and are vulnerable to various injuries, especially hypoxia insult (38). We used an *in vitro* cell model that has been widely used to simulate renal IRI by harvesting the culture supernatant (termed conditioned medium, CM) from a mouse renal TEC cell line (mRTECs) after hypoxia-reoxygenation (H/R) injury (39–41). Bone marrow-derived macrophages (BMDMs) from WT and Lyz-TSC1 cKO mice were induced by M-CSF stimulation and then treated with CM for 48 h (**Figure 5A**). The treated BMDMs in each group were collected for a flow cytometry analysis. The data show that after the treatment with CM, the *TSC1* cKO BMDMs expressed significantly lower levels of CD206 than the WT BMDMs (**Figures 5B, C**). The transcriptional level of gene expression was also determined by real-time PCR. The hallmarks of M2 macrophages, including CD206 and Arginase-1 (Arg-1), showed significantly lower expression in the *TSC1* cKO macrophages. Moreover, the expression of C/EBPβ in the *TSC1* cKO BMDMs was significantly lower than that in the WT BMDMs (**Figure 5D**). The rapamycin treatment for 48 h markedly reversed the damaged C/EBPβ expression in the *TSC1* cKO macrophages (**Figure 5E**). These findings demonstrate that the decreased C/EBPβ expression in the *TSC1* cKO macrophages may also be induced by overactivated mTOR activity, which is consistent with our previous study (24).

### The Decreased C/EBPβ Expression in the *TSC1* cKO Macrophages During the Repair Phase of IRI May Also Be Induced by Overactivated mTOR Activity

**Figure 6:**
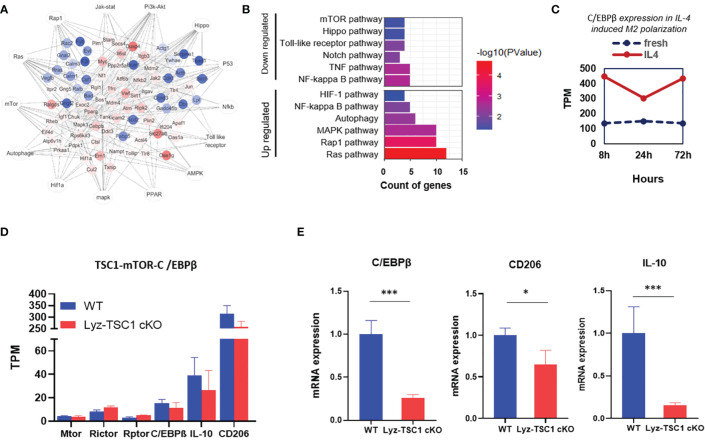
Decreased C/EBPβ expression in *TSC1* cKO renal macrophages during the repair phase of IRI may also be induced by overactivated mTOR activity. **(A)** Network summary of previous enrichment of the KEGG pathway related to M2 polarization. **(B)** Top 12 most up- and downregulated pathways by differential mRNA. **(C)** C/EBPβ expression with or without IL-4 induced M2 polarization in the downloaded data. **(D)** TPM value of mTOR, Rictor, Raptor, C/EBPβ, and IL-10 in macrophages from WT or Lyz-TSC1 cKO mouse kidneys 2 weeks after ischemia-reperfusion. **(E)** Relative expression of C/EBPβ, CD206, and IL-10 in kidney macrophages sorted from WT and Lyz-TSC1 cKO mice on day 14 post-IRI. In **(E)**, the data are shown as the mean ± SD and were analyzed by an unpaired two-tailed Student’s t-test (n ≥ 3). *P < 0.05; **P < 0.01; NS, not significant.

To further verify the conclusions drawn in the previous section, we sorted the WT and Lyz-TSC1 cKO mouse kidney macrophages (CD45^+^F4/80^+^CD11b^+^) 2 weeks after reperfusion and performed RNA sequencing. According to previous reports, we summarized the previous enrichment of the KEGG pathway related to M2 polarization (**Figure 6A**). Accordingly, we enriched the related top 12 of the most up- and downregulated pathways by the differential mRNAs (**Figure 6B**). We further selected the gene set enrichment analysis (GSEA) results of the top M2 polarization-related items in **Supplementary Figure 4**, including the autophagy signaling pathway, NF-kb signaling pathway, PI3K-Akt signaling pathway, and Notch signaling pathway. However, regarding these pathways, the WT group showed a commonly lower enrichment score (ES) than the Lyz-TSC1 cKO group. By analyzing the data in the GEO database (GEO accession GSE107776), we found that during the M2 polarization process induced by IL-4, the expression of C/EBPβ was increased compared to that in the control group (**Figure 6C**), which is consistent with the results of the previous regulation of M2 polarization observed in our laboratory (24). Our sequencing data demonstrated that the expression of Raptor and Rictor, which are unique subunits in mTORC1 and mTORC2, respectively, was upregulated in the Lyz-TSC1 cKO group; The Lyz-TSC1 cKO group expressed lower levels of C/EBPβ than the WT group (**Figure 6D**). Larabee JL’s study demonstrated that C/EBPβ binding was detected in the promoter of the IL-10 gene in macrophages, which is necessary for the induction of IL-10 (42). Our sequencing data confirmed that the expression of IL-10 was consistent with the expression of C/EBPβ in the two groups. Then, the WT and Lyz-TSC1 cKO mouse kidney macrophages (CD45^+^F4/80^+^CD11b^+^) were sorted 2 weeks after reperfusion, and we performed real-time quantitative PCR. The macrophages from the Lyz-TSC1 cKO mouse group expressed decreased C/EBPβ, CD206, and IL-10, which is consistent with the sequencing data (**Figures 6D, E**). These data support our *in vitro* study and indicate that the M2 polarization deficiency of the TSC1KO renal macrophages after ischemia-reperfusion may also be mediated by the mTOR-C/EBPβ pathway.

## Discussion

We previously reported that TSC1 is an important regulator of M1 and M2 activation of macrophages (24). In this study, we found that during the early phase after renal ischemia-reperfusion, the elevated injury in the Lyz-TSC1 cKO mice was related to increased M1 polarization of *TSC1* cKO renal macrophages. We also demonstrated that during the repair process of renal IRI, the depletion of *TSC1* in macrophages decreased renal fibrosis by preventing M2 polarization. Collectively, TSC1 affects the overall process of ischemia-reperfusion by regulating the polarization of macrophages.

Our results show an aggravated trend of renal injury in Lyz-TSC1 cKO mice during the early stage after renal ischemia-reperfusion, including higher renal tubular injury scores, higher serum creatinine levels, and increased KIM-1 levels. We also found that elevated injury may be caused by increased M1 polarization in TSC1 knockout macrophages during the early phase rather than an increased number of infiltrated leukocytes or macrophages by RNA sequencing, flow cytometry analysis, adoptive transfer, and quantitative PCR. Our previous study and the work of others demonstrated that Lyz-TSC1 cKO mice exhibited an enhanced M1 response when treated with LPS *in vivo* or *in vitro* in an mTOR-independent or mTOR-dependent pathway (24, 43). LPS induces an acute endotoxin shock model, which is a type of septic shock (44). Therefore, we first proved that *TSC1* deficiency in macrophages may promote M1 polarization to enhance inflammation and aggravate early tubular epithelial injury after ischemia-reperfusion during the early phase of ischemia-reperfusion injury, i.e., sterile inflammation. However, whether mTOR is dependent in this study needs further exploration.

During the recovery and repair phase of postischemia/reperfusion injury, we found that the renal interstitial fibrotic and cellulosic area in the Lyz-TSC1 cKO mice was decreased compared with that in the WT group. M2 macrophages perform wound healing and play profibrotic roles during renal repair (21, 45). As mentioned above, macrophages (especially the CD206^+^ subset of M2 macrophages) are strongly associated with renal fibrosis (5). Therefore, we hypothesized that the depletion of *TSC1* in macrophages would reduce fibrosis by blocking M2 polarization after ischemia-reperfusion. First, we verified that *TSC1* cKO macrophages are refractory to M2 polarization, which was confirmed by a flow cytometry analysis of M2-like macrophages (F4/80highCD11b+ cells) and CD206 expression 7, 14, and 21 days after reperfusion. The coimmunostaining of immunofluorescence with F4/80 and CD206 14 days after reperfusion also supported the above results. Reduced co-expressing of CD206 and α-SMA in Lyz-TSC1 cKO group suggests lower numbers of MMT cells and reduced fibrosis compared with WT group. Our previous study indicated that the reduced C/EBPβ expression in *TSC1* cKO macrophages is induced by overactivated mTOR activity and is involved in poor M2 polarization (24). Similar to the *in vivo* results, the *TSC1* cKO BMDMs demonstrated a decreased M2 polarization trend compared to the WT BMDMs after the treatment with CM *in vitro*, which is a cell model that has been widely used to study IRI (39–41). The expression of C/EBPβ in the *TSC1* cKO macrophages was significantly lower than that in the WT macrophages, while the Rapa treatment reversed the expression of C/EBPβ by *TSC1* cKO macrophages, which is consistent with previous reports. To verify this finding, kidney macrophages (CD45^+^F4/80^+^CD11b^+^) from WT and Lyz-TSC1 cKO mice were sorted for RNA sequencing and real-time quantitative PCR 2 weeks after reperfusion. Both results demonstrated that the macrophages from the Lyz-TSC1 cKO mice also expressed decreased C/EBP-β.

A previous study found that the 38 kDa isoform of C/EBPβ is indispensable for the expression of IL-10 (42). Consistent with this observation, our study demonstrated that the expression of IL-10 in Lyz-TSC1 cKO kidney macrophages was reduced based on sequencing data and real-time quantitative PCR. As previously noted, long-term IL-10 exposure or IL-10 application to chronic disease processes may promote fibrotic results (12, 13, 46, 47). Therefore, these findings may indicate that C/EBPβ has multiple regulatory effects on renal fibrosis during the recovery and repair phases. On the one hand, decreased C/EBPβ expression in *TSC1* cKO macrophages is involved in poor M2 polarization, which is strongly associated with renal fibrosis. On the other hand, a decrease in IL-10 may lead to weakened subsequent tubule interstitial fibrosis by reducing C/EBPβ expression. In addition, studies have shown that the chronic fibrogenic process is detrimental to the nephron and surrounding vasculature (48–50), which might explain why the Lyz-TSC1 cKO mice showed aggravated kidney dysfunction (tubular damage and serum creatinine) after reperfusion during the early phase; however, surprisingly, during renal repair, kidney dysfunction did not continue to deteriorate, and no significant difference was observed between the groups. Besides, we did not conduct *in vivo* experiments using Rapa to reverse the fibrosis reduction because under pathological conditions, the mTOR activities of numerous fibroblasts in mouse kidneys are also upregulated (51, 52). Therefore, the combined effects of Rapa on overall fibrosis of the kidney are mostly considered to have an improved effect (53, 54). Guochun Chen’s study demonstrated that Rapa ameliorates kidney fibrosis by inhibiting the activation of mTOR signaling in interstitial macrophages (55). However, this conclusion is based on a report suggesting that inflammatory cell infiltration is an early feature of renal fibrosis in almost all cases (56); thus, the role of macrophage subpopulations was not considered because macrophages exhibit completely different phenotypes during kidney injury and repair.

In summary, this study demonstrates that *TSC1* deficiency in macrophages may promote M1 polarization to aggravate kidney dysfunction after ischemia-reperfusion during the early phase, while during the repair process of ischemia-reperfusion, *TSC1* deficiency reduced M2 macrophage polarization, leading to decreased renal fibrosis. The findings presented in this study provide new insight into therapeutic targeting in sterile renal injury and kidney fibrosis during the transition from AKI to CKD.

## Data Availability Statement

The datasets presented in this study can be found in online repositories. The names of the repository/repositories and accession number(s) can be found below: http://www.ncbi.nlm.nih.gov, PRJNA679157.

## Ethics Statement

The animal study was reviewed and approved by the research ethics committee (IRB: [2020]02-166) at the third affiliated Hospital of Sun Yat-sen University.

## Author Contributions

NN and YZ conceived the project. XH, YX, ZZ, ZT, JZ, YL, WD, and ZD designed the experiments. XH and YX conducted the experiments. ZZ performed the sequencing and statistical analyses. XH, YX, and ZZ wrote the manuscript. All authors contributed to the article and approved the submitted version.

## Funding

This research was supported by the National Natural Science Foundation of China (No. 81970652 and No. 81470977), the Natural Science Foundation of Guangdong Province (No. 2019A1515011219), the Bioengineering Research Center Training Project of the Third Affiliated Hospital of Sun Yat-sen University (SW201904), and the Science and Technology Planning Project of Guangzhou (No. 201803010016).

## Conflict of Interest

The authors declare that the research was conducted in the absence of any commercial or financial relationships that could be construed as potential conflicts of interest.
