# Supplementary material for: TSC1 Affects the Process of Renal Ischemia-Reperfusion Injury by Controlling Macrophage Polarization
Source: Front Immunol. 2021 Mar 9;12:637335. doi: 10.3389/fimmu.2021.637335 (PMC7985265; doi:10.3389/fimmu.2021.637335)
Supplement: Supplementary file 1 [file DataSheet_1.docx]

| **NAME** | **type** | **WT-average** | **KO-average** |
| --- | --- | --- | --- |
| Il1a | Down | 19.42728 | 13.91918 |
| Il23a | Down | 2.993363 | 0.165251 |
| Il1b | Up | 1599.056 | 1998.474 |
| Il6 | Up | 5.303594 | 6.035442 |
| Il27 | Up | 21.97024 | 24.0404 |
| Tnf | Up | 515.7033 | 679.6751 |
| Acsf3 | Up | 0.926545 | 1.462041 |
| Nfkb1 | Up | 16.84323 | 28.41944 |
| Nfkb2 | Up | 3.365535 | 3.639938 |
| Nfkbia | Up | 609.3964 | 775.196 |
| Nfkbib | Up | 18.14559 | 41.54307 |
| Nfkbid | Up | 48.99703 | 69.35703 |
| Nfkbie | Up | 27.27565 | 39.37367 |
| Nfkbil1 | Up | 2.006417 | 10.64123 |
| Nfkbiz | Up | 93.10503 | 170.0591 |
| Cxcl2 | Up | 903.7327 | 988.1211 |
| Tnfaip3 | Up | 35.61063 | 54.77061 |
| Socs3 | Up | 95.05053 | 117.8629 |
| Peli1 | Up | 24.88807 | 37.87545 |

**Supplemental Table 1**

Expression of M1 related genes

**Supplemental Table 2**

The primer used in the RT-PCR

| **Primer** | Sequence (5'-3') |
| --- | --- |
| **TNF-α** | F:CTTCTGTCTACTGAACTTCGGG |
|  | R:CAGGCTTGTCACTCGAATTTTG |
| **iNOS** | F:CACCAAGCTGAACTTGAGCG |
|  | R:CGTGGCTTTGGGCTCCTC |
| **IL-1β** | F:ACGGACCCCAAAAGATGAAG |
|  | R:TTCTCCACAGCCACAATGAG |
| **IL-10** | F:AGCCGGGAAGACAATAACTG |
|  | R:GGAGTCGGTTAGCAGTATGTTG |
| **TGF-β** | F:CCTGAGTGGCTGTCTTTTGA |
|  | R:CGTGGAGTTTGTTATCTTTGCTG |
| **CD206** | F:TTGGACGGATAGATGGAGGG |
|  | R:CCAGGCAGTTGAGGAGGTTC |
| **Arg-1** | F:AAGAATGGAAGAGTCAGTGTGG |
|  | R:GGGAGTGTTGATGTCAGTGTG |
| **CEBP-β** | F:GTTTCGGGACTTGATGCAATC |
|  | R:TTTAAGGTGATTACTCAGGGCC |
| **HPRT** | F: AGTACAGCCCCAAAATGGTTAAG |
|  | R: CTTAGGCTTTGTATTTGGCTTTTC |


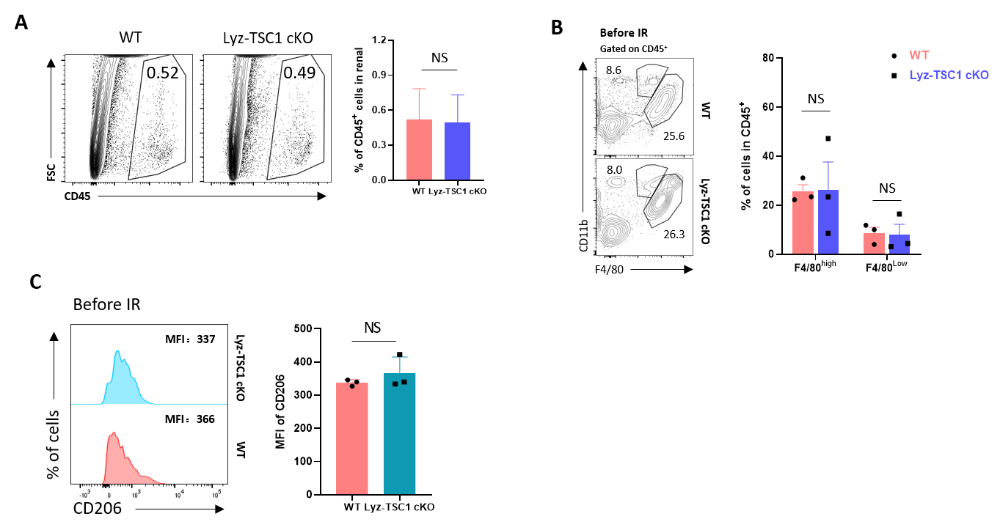


Supplementary Figure 1 Normal proportion of immune cells and macrophages phenotype in renal of Lyz-TSC1 cKO mouse in steady-state. (A) The levels of renal leukocytes(CD45^+^) in WT and Lyz-TSC1 cKO mice before the ischemia-reperfusion injury. (B) The levels of F4/80^high^ macrophages and the ratio of F4/80^high^ to F4/80^low^ macrophages in WT and Lyz-TSC1 cKO mice before the ischemia-reperfusion injury. (C) CD206 mean fluorescence intensity (MFI) of macrophages in WT and Lyz-TSC1 cKO mice kidneys before the ischemia-reperfusion injury. For (A),(B),(C), data are shown as mean ± SD and were analyzed by Unpaired two-tailed Student's t-test (n≥3)*, P<0.05; **, P<0.01; NS, not significant.


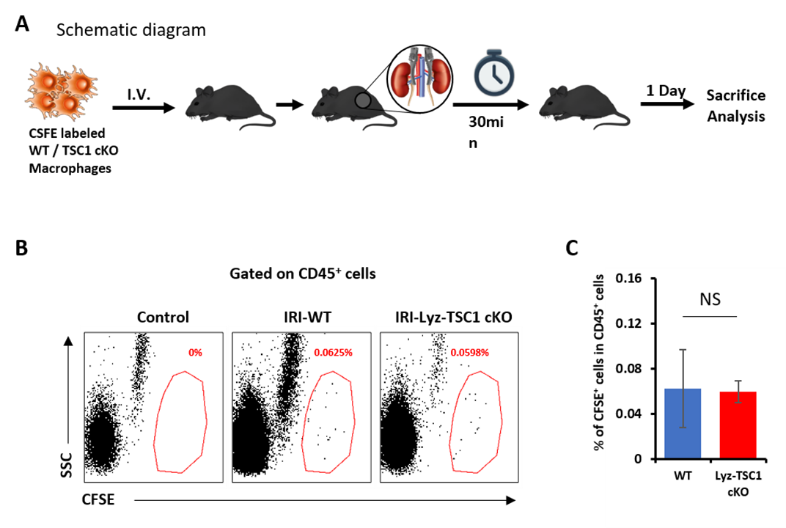


Supplementary Figure 2 CFSE-labeled WT or Lyz-TSC1 cKO BMDMs were adoptively transferred into WT recipients before kidney IRI. (A) Schematic diagram. (B) Representative images of CFSE-labeled WT and Lyz-TSC1 cKO BMDMs that infiltrated into the kidneys, gating on CD45^+^. (C) Quantification of the proportion of CFSE^+^ cells in CD45^+^ cells. For (C), data are shown as mean ± SD and were analyzed by Unpaired two-tailed Student's t-test (n＝3)*, P<0.05; **, P<0.01; NS, not significant.


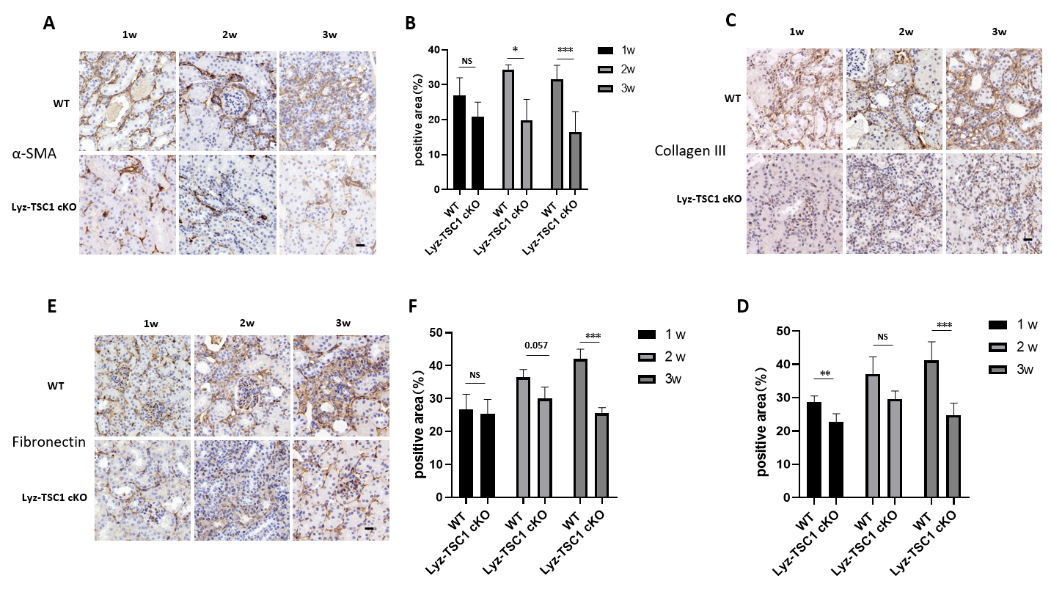


Supplementary Figure 3 Representative micrographs show a-smooth muscle actin (a-SMA), Collagen III, and fibronectin expression 7,14,21 days after IRI the WT and Lyz-TSC1 cKO groups.(A) Representative a-SMA immunohistochemistry in renal sections from WT and KO mice 7,14,21 days after IRI . Scale bar: 20 µm. (B) Quantification of a-SMA immunohistochemistry-stained murine renal cortical sections on day 7, day 14, and day 21 after IRI. (C), (D) Representative immunohistochemistry and quantification of a-SMA. (E), (F) Representative immunohistochemistry and quantification of fibronectin. For (B),(D),(F), data are shown as mean ± SD and were analyzed by Unpaired two-tailed Student‘s t-test (n≥3)*, P<0.05; **, P<0.01; ***, P<0.001; NS, not significant.


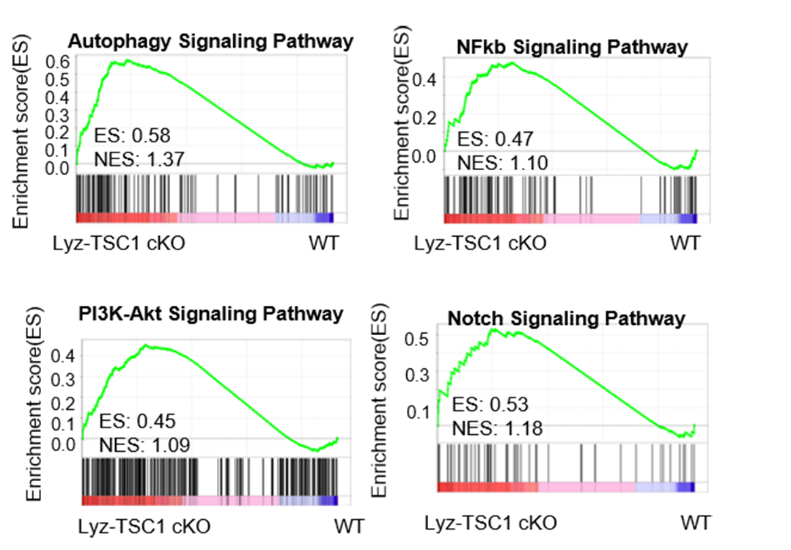


Supplementary Figure 4 The GSEA results of the top M2-polarization-related items, including the Autophagy Signaling Pathway, NF-kb Signaling Pathway, PI3K-Akt Signaling Pathway, and Notch Signaling pathway.
